# The Relationship Between the Modulation of Intestinal Microbiota and the Response to Immunotherapy in Patients with Cancer

**DOI:** 10.3390/biomedicines13010096

**Published:** 2025-01-03

**Authors:** Laura Radoš, Marin Golčić, Ivana Mikolašević

**Affiliations:** 1 Department for Emergency Medicine of Primorsko-Goranska County, 51000 Rijeka, Croatia; laura1301rados@gmail.com; 2 Clinic for Tumors, Clinical Hospital Center Rijeka, 51000 Rijeka, Croatia; ivana.mikolasevic@medri.uniri.hr; 3 School of Medicine, University of Rijeka, 51000 Rijeka, Croatia

**Keywords:** immunotherapy, fecal microbial transplantation, microbiome, antibiotics, probiotics, diet

## Abstract

The intestinal microbiota is an important part of the human body, and its composition can affect the effectiveness of immunotherapy. In the last few years, the modulation of intestinal microbiota in order to improve the effectiveness of immunotherapy has become a current topic in the scientific community, but there is a lack of research in this area. In this review, the goal was to analyze the current relevant literature related to the modulation of intestinal microbiota and the effectiveness of immunotherapy in the treatment of cancer. The effects of antibiotics, probiotics, diet, and fecal microbial transplantation were analyzed separately. It was concluded that the use of antibiotics, especially broad-spectrum types or larger quantities, causes dysbiosis of the intestinal microbiota, which can reduce the effectiveness of immunotherapy. While dysbiosis could be repaired by probiotics and thus improve the effectiveness of immunotherapy, the use of commercial probiotics without evidence of intestinal dysbiosis has not yet been sufficiently tested to confirm its safety for cancer for immunotherapy-treated cancer patients. A diet consisting of sufficient amounts of fiber, as well as a diet with higher salt content positively correlates with the success of immunotherapy. Fecal transplantation is a safe and realistic adjuvant option for the treatment of cancer patients with immunotherapy, but more clinical trials are necessary. Modulating the microbiota composition indeed changes the effectiveness of immunotherapy, but in the future, more human studies should be organized to precisely determine the types and procedures of microbiota modulation.

## 1. Introduction

The human intestinal microbiota consists of bacteria, fungi, protozoa, archaea, and viruses, and plays a significant role in preserving the structural, metabolic, neurological, and immunological homeostasis of the human body [1]. Intestinal functions of microbiota are divided into structural, metabolic, neurological, and protective [1]. Recent data also shows a complex and bidirectional relationship between microbiota and cancer [2]. 

The connection between the immune system and malignant diseases was established in 1863, when Rupert Virchow first proposed the idea of a cascade from inflammation to cancer. However, over a hundred years passed before discovering that immunoglobulins cytotoxic-T-lymphocyte-associated protein (CTLA-4) and programmed cell death 1 (PD-1) function as inhibitors of T lymphocyte activation, and they have since become the key targets of modern immunotherapy [3]. Anti-PD1 and anti-CTLA-4 antibodies have significantly increased the survival of patients with advanced cancers, such as metastatic melanoma, whose 5-year survival in 2009 was around 10% [4], while today, it is possible to achieve a 5-year survival of 52% [5]. Despite the significant advances that immunotherapy has brought to modern cancer treatment, most patients disease will still progress on immunotherapy, and it is necessary to find a way to increase its effectiveness [6]. In this review, we will evaluate the potential of enhancing the response to immunotherapy through the modulation of the intestinal microbiome.

### 1.1. Intestinal Microbiota Composition

**Figure 1:**
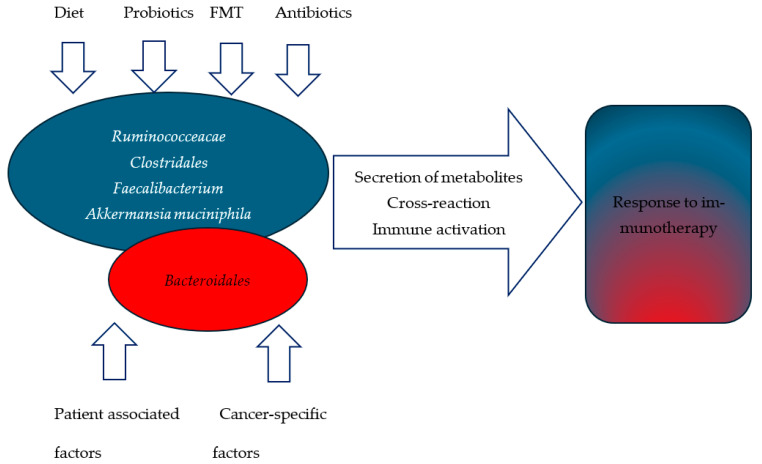
The variable association between different bacteria and response to immunotherapy with potential factors influencing the response. The blue color indicates bacteria genus, order, and species associated with a good response to immunotherapy.

The composition of the intestinal microbiota of each person is kept in relative homeostasis, but it is variable depending on factors such as diet, environment, lifestyle, diseases, and drugs, especially antibiotics [1]. Modern research on the relationship between intestinal microbiota and immunotherapy began in 2015, when it was noticed that mice with sterile guts exhibit a worse clinical response to cancer immunotherapy treatment than mice with a more diverse gut microbiome [7]. However, several subsequent studies have shown heterogeneous results related to the exact composition of the microbiota that promotes the effectiveness of immunotherapy, and the results only partially overlap. The majority of studies highlight the genus *Ruminococceacae* [8,9,10,11,12], order *Clostridales* [9,10,13], genus *Faecalibacterium* [9,12,13], and the bacterium *Akkermansia muciniphila* [10,14,15,16,17] as more represented in the groups that achieved a clinical response to immunotherapy (responder group—R). On the other hand, it was shown that *Bacteroidales* order was more represented in the groups of patients who did not achieve a clinical response (non-responder group—NR) [8,9,11,12,18]. One of the ways in which the microbiota can affect the effect of immunotherapy is through the secretion of metabolites of inosine, tryptophan, metabolites of bile acids, and short-chain fatty acids, which affect genetic and epigenetic regulation, tumor signaling pathways, DNA repair, and the metabolism of cells of the immune system [19]. Metabolites can be pro-tumorigenic, anti-tumorigenic, or even both, depending on the environment in which it is found inside the body [2,19]. Via the cross-reaction between the gut microbiota and the cells of the immune system, the intermediate functions of innate immune cells increase, the anti-tumor effect of adaptive immune cells strengthens and the immunogenicity of tumor cells amplifies. In this way, the intestinal microbiota can reprogram the tumor microenvironment and enhance the response to immunotherapy [20] (Figure 1).

### 1.2. Immunotherapy

Some of the FDA-approved immunotherapy drugs include pembrolizumab, nivolumab, cemiplimab, atezolizumab, durvalumab, avelumab, and ipilimumab [6]. Various cancers are treated with immunotherapy and include melanoma, squamous cell skin cancer, Merkel cell cancer, head and neck cancers, anal cancer, lung cancer, colorectal cancer, gastric and esophageal cancer, cervical cancer, kidney cancer, pleural mesothelioma, urothelial cancer, and hepatocellular carcinoma, although this list is expected to expand [6,21]. Immunotherapy can be accompanied by numerous side effects, which are classified as low-grade (grades 1 and 2) and high-grade (grades 3 and 4). Side effects are most often dermatological, gastrointestinal, endocrine, pulmonary, rheumatological, neurological, ocular, renal, cardiac, and hematological, but any organ in the body can be affected [21]. Side effects of grade 3 or more are more common in treatment with anti-CTLA-4 immunotherapy (up to 31%), while in treatment with anti-PD-1, they occur in up to 10% of cases [22].

### 1.3. Antibiotics and Immunotherapy

Antibiotics (ABs) affect the intestinal microbiota by causing dysbiosis. They change the composition of the microbiota, reducing the diversity and richness of the composition, decreasing the abundance of some species, and increasing the abundance of others, depending on the AB itself and the breadth of its spectrum of action. The breadth of an AB’s spectrum refers to the range of bacteria an antibiotic can effectively target, which can be narrow or broad. The broader the AB spectrum is, the more significant effects on the composition of the intestinal microbiota occur [23].

Several manuscripts evaluated the effect of AB on immunotherapy in cancer patients. Derosa et al. studied the effect of taking AB before the start of immunotherapy on patients with non-small-cell lung cancer (NSCLC) and renal cell carcinoma (RCC). The most common ABs were beta-lactams. Both RCC and NSCLC patients showed shorter progression-free survival (PFS) and overall survival (OS) versus patients who did not receive AB [24].

Similarly, Pinato et al. also demonstrated that OS in patients with different cancer types who received AB before starting immunotherapy was significantly lower compared to those who did not receive AB, and the chance of worse response to immunotherapy treatment was almost twice as high compared to those who did not receive AB. The beta-lactams, which were the most frequently prescribed AB, did not seem to affect survival if they were used during immunotherapy [25].

Similar results were shown for melanoma patients as well. Elkrief et al. showed that beta-lactams were the most common AB in their patient cohort, with shorter PFS and lower objective response (OR) (0 vs. 34%) for patients receiving AB [26]. On the other hand, cephalosporins, penicillins, and fluoroquinolones were the most common AB in melanoma cohort evaluated by Mohiuddin et al. However, the effect was similar: the AB cohort had a significantly shorter OS, regardless of whether ABs were used 45 or 90 days before immunotherapy. Furthermore, patients treated with ABs had a higher occurrence of immunotherapy-related colitis requiring intravenous steroids in the AB cohort [27].

As patients use not only different ABs but also use the same AB for different durations; it is important to try to quantify this as well. Geum et al. also studied the impact of AB both 30 days before the start and during immunotherapy in NSCLC patients but additionally evaluated the impact of the dose and type of AB used. Patients receiving AB showed significantly shorter OS but not PFS compared to those who did not receive AB, while broad-spectrum piperacillin/tazobactam showed both shorter OS and shorter PFS compared to patients who did not receive AB. This study also quantified AB intake through two measures: the sum of antibiotic days of therapy (DOT) and the defined daily dose of AB (DDD). Higher DOT correlated with lower OS in all ABs and lower PFS in taking piperacillin/tazobactam longer than 2 weeks, while higher DDD of piperacillin/tazobactam and glycopeptides correlated with higher mortality. This study suggested that broad-spectrum ABs and higher cumulative doses have a more profound effect on survival [28].

Additionally, Iglesias-Santamaria et al. tried to calculate the measure of exposure to AB (AE): the ratio of the number of days the patient received AB to the number of days they were treated with immunotherapy. The most used ABs were beta-lactams, followed by fluoroquinolones, cephalosporins, and macrolides. The research group did not find a difference in PFS and OS regardless of whether ABs were used before or after the start of immunotherapy. However, the difference in PFS and OS was significant when comparing patients who had an AE greater than the median (which was 11.1%) with those who had an AE at or below the median, demonstrating the negative impact of a significant AE. The study suggested that the length of treatment with ABs is more important than the timing [29].

While not evaluating AE, Tinsley et al. showed a detrimental effect on PFS and OS for melanoma, NSCLC, or RCC patients who received AB for more than 7 days compared to those who did not or who received only one dose of AB in all three tumor types [30].

Additionally, an interesting observation of ABs’ detrimental effect was demonstrated by Glitza et al. The randomized study aimed to evaluate the effectiveness of immunotherapy combined with SER-401, a formulation of Firmicutes spores fractioned and purified from human stool, in comparison to just placebo and immunotherapy. As a mean of preparation for receiving SER-401, the research applied vancomycin; however, such application was found to be negatively associated with patient survival [31].

Routy et al.’s preclinical work provides support for these clinical observations. Their murine model demonstrated that broad-spectrum ABs disrupted the antitumor efficacy of immunotherapy, likely through microbiota-dependent pathways. Their findings from the murine model were also shown to be true for patients with NSCLC, RCC, and urothelial carcinoma, where AB use was associated with shorter PFS and OS [17] (Table 1).

### 1.4. Probiotics, Dietary Habits, and Immunotherapy

#### 1.4.1. Probiotics

The roots of probiotics usage for microbiota modulation date all the way back to ancient China [32]. According to the definition of the World Health Organization, probiotics are products consisting of live microorganisms that, when given in appropriate amounts, provide health benefits to the host [33]. The first available probiotics, at the end of the 1990s, consisted mostly of *Saccharomyces* or *Lactobacillus* strains, which were considered useful in preventing infectious diarrhea or *Clostridium difficile* infection after using AB. Probiotics are highly variable between themselves, often contain multiple strains, and usually consist of 10^8^ to 10^10^ organisms per capsule or sachet. They often do not require the same regulations as prescription medicines. This makes it difficult to compare the products or adequately study the effectiveness of individual types of strains [32]. Current research shows that certain probiotics and prebiotics help in the prevention and treatment of certain diseases, but also that it is necessary to conduct more research on them and to clinically validate more probiotics and prebiotics, considering the size of the market and the various compositions of individual products [34].

Despite the widespread use of probiotics, the number of studies evaluating the effectiveness of probiotics for patients on immunotherapy is very scarce. Tomita et al. retrospectively studied the effect of *Clostridium butyricum* probiotics on NSCLC patients, of which some used only probiotics and some used both AB and the probiotic *Clostridium butyricum*. Patients who received probiotics in addition to AB had significantly better PFS and OS compared to patients who received AB alone. This study showed that the probiotic *Clostridium butyricum* has a significant role in the survival of patients who received AB and a less significant role in the survival of patients who did not receive AB [35]. Dizman et al. prospectively evaluated the impact of *Clostridium butyricum* on RCC patients receiving the nivolumab–ipilimumab, confirming the results of Tomita et al. and showing a significantly longer PFS in patients receiving the probiotic. However, OS was not studied because the last analysis was conducted 12 weeks after the first therapy [36]. Positive results in a murine model were also found for *Lactisaeibacillus rhamnosus* (LGG) probiotics in a trial by Gao et al. [37]. Cohort treated with probiotics along with anti-PD-1exhibited a higher abundance of bacteria associated with short-chain fatty acids (SCFA), which are associated with a positive antitumor response [18]. However, this study was not continued in human patients.

One of the few trials in humans includes the work by Takada et al., who focused on NSCLC patients who received anti-PD-1 monotherapy, some of whom used probiotics *Bifidobacterium*, *Clostridium butyricum*, and AB-resistant lactic acid bacteria. The study showed that those patients who received probiotics had a longer PFS than patients who did not receive probiotics, but there was no significant difference in OS. It is important to emphasize the imbalance in the number of patients with and without probiotics, which could have affected the results [38]. Unlike those trials, Spencer et al. evaluated the effects of both diet and probiotics and demonstrated that the group of patients who did not use probiotics but used enough fiber exhibited the longest survival, demonstrating a potentially detrimental effect of unselected use of probiotics on the effectiveness of immunotherapy. Their hypothesis was tested in mice with melanoma who were treated with FMT from a CR patient, with one group additionally receiving either the probiotic *Bifidobacterium longum* or the probiotic LGG. Mice that received the probiotic had a significantly lower amount of CD8+ cells in the tumor [39].

#### 1.4.2. Diet

Diet, and particularly dietary fibers are important sources of energy for the intestinal microbiota. They are positively associated with micronutrient availability, stool formation, and microbial specificity. Food enriched with fiber has been shown to be an important factor in the prevention and treatment of many gastrointestinal disorders [40]. As previously mentioned, the research by Spencer et al. demonstrated that dietary fibers are important for a successful response to immunotherapy. Patients who used sufficient fiber (20 g per day), especially when they did not additionally use unselected probiotics, reported the longest PFS. To confirm the role of dietary fiber, normal-bred melanoma mice treated with immunotherapy were fed high-fiber and low-fiber diets, with the cohort treated with high-fiber content reporting slower tumor growth. There was no such difference in mice with sterile intestines (germ-free mice), which is a proof that the influence of the fiber diet depends on the composition of microbiota [39]. The value of diet during immunotherapy was confirmed by Lam et al., who showed that mice fed with a high-fiber diet showed slower tumor progression and greater activation of intratumoral dendritic cells compared to mice fed a Western-type diet (a diet high in fat and sugar). This study showed that a high-fiber diet enriches the bacteria *Akkermansia municiphila*, which promotes the activation of NK cells and dendritic cells [15].

The potential link between diet and response to immunotherapy in melanoma patients was also shown by Simpson et al. The study included patients from the Netherlands, Australia, and the USA. Clinical responses were better in patients with gut microbiota dominated by *Ruminococcaceae* versus those dominated by *Bacteroidaceae*. *Ruminococcaceae* abundance was associated with higher microbiome diversity, higher fiber intake, and lower baseline C-reactive protein (CRP), and *Bacteroidaceae* with lower microbiome diversity, lower fiber intake, and higher baseline CRP. Patients from Australia and the USA, where the Western diet prevails, had the majority of microbiota compositions dominated by *Bacteroidaceae*, while patients from the Netherlands, where the high-fiber diet prevails, had the majority of microbiota compositions dominated by *Ruminococcaceae* [11].

Salt seems to be another important dietary factor, as Rizvi et al. demonstrated. The study comprised three groups of mice: a low-salt diet (LSD), a normal diet (ND, normal amount of salt), and a high-salt diet (HSD) cohort. The HSD group showed higher activity of NK cells and interferon gamma (IFNγ), which are important for the intratumor immune response [9], and had significantly less tumor progression and significantly higher survival than the LSD and ND groups. A higher amount of serum hippuric acid was observed, which could be useful in anti-PD-1 efficacy, and microbiota analysis showed a higher amount of *Bifidobacterium* spp. FMT from the HSD group was performed on a new group of mice, and this group showed a 42% tumor regression compared to those who received FMT from the NT group of mice. It was concluded that HSD counteracted hyponatremia, which is common in cancer patients, and enriched *Bifidobacterium*, which could influence better tumor immunity [41].

Data on probiotics and dietary habits is summarized in Table 2.

### 1.5. Immunotherapy and Fecal Microbiota Transplantation

FMT is a process by which fecal material is transferred from the donor to the recipient, and it can be performed via nasogastric tube, nasojejunal tube, endoscopy, colonoscopy, or in the form of an oral capsule. FMT is used for the treatment of recurrent infections with the bacterium *Clostridiun difficile* and is also being investigated as a treatment option for ulcerative colitis, irritable bowel syndrome, and other gastrointestinal diseases. FMT is not without risks, including the unwanted introduction of pathogens [2].

The first attempts at FMT related to the effectiveness of immunotherapy treatment were made in 2015 in mice. Sivan et al. observed different melanoma growth in genetically identical mice from two different institutions. They divided the mice from the two institutions into groups named JAX and TAC. The TAC group showed more aggressive melanoma growth compared to the TAC group, and it was assumed that the cause was an external factor. TAC mice were placed in the same environment as the JAX group, and it was observed that the melanoma in this case grew at a similar rate. Assuming that the composition of the microbiota is the cause of the difference between melanoma growth in the two groups, the TAC group was treated with FMT from the JAX group, resulting in slower tumor growth in the recipient mice and infiltration of antigen-specific T cells into the tumor. Microbiota analysis revealed that *Bifidobacterium* spp. was associated with a significant antitumor response, specifically one taxon most similar to *B. breve*, *B. longum*, and *B. adolescentis* [16]. Additionally, an FMT trial in mice was conducted by Routy et al. after studying the correlation of microbiota composition in 60 patients with NSCLC and 40 patients with RCC. In both the RCC and NSCLC groups, a correlation with a positive clinical response was observed in patients with abundant *Akkermansia muciniphila*. Specifically, in NSCLC R patients, there was a higher abundance of *Ruminococcus* spp., *Alistipes* spp., and *Eubacterium* spp., with a lower abundance of *Bifidobacterium adolescentis*, *B. longum*, and *Parabacteroides distasonis*. The researchers performed FMT from both R and NR patients onto mice. Mice receiving FMT from the R group exhibited reduced tumor growth and a more significant response to immunotherapy; a similar result was achieved in mice that received only *A. municiphila* [17]. Similar results were achieved by Gopalakrishnan et al. who showed that mice that received FMT from the R group of melanoma patients, exhibited more *Faecalibacterium* and greater infiltration of CD8+ cells into the tumor and fewer suppressive myeloid cells compared to mice that received FMT from NR donors. *Ruminococcaceae* families were found in the microbiota in the R group, of which *Faecalibacterium* correlated with significantly longer PFS, while a greater abundance of *Bacteroidales* was reported in the NR group [8].

After FMT was shown to be safe and successful in mice, the first human study of FMT and immunotherapy was conducted by Baruch et al. in 2020 on 10 patients with anti-PD-1 refractory metastatic melanoma, examining the safety and efficacy of FMT in the treatment of metastatic melanoma with anti-PD-1. Two donors were selected to provide the sample, both patients who achieved an objective clinical response lasting more than a year after treating metastatic melanoma with anti-PD-1. FMT was initially given colonoscopically and in the form of oral capsules, followed by an oral capsule every 14 days with an anti-PD-1 cycle. Responders were defined as patients who achieved reduction of the tumor mass by at least 30%, and this was shown in three patients, of which two patients had a partial response (PR), and one had a CR to therapy. All patients had PFS longer than 6 months, and they were recipients of the first donor. All five recipients of the second donor and two recipients of the first donor were NR. Recipients of the first donor had a higher relative amount of *Bifidobacterium adolescentis*, and recipients of the second donor had a higher relative amount of *Ruminococcus bromii*. The composition of the R and NR microbiota within the first donor group did not differ significantly either functionally or metabolically. All five recipients showed intestinal infiltration with antigen-presenting cells, and the intratumor showed increased regulation of genes for IFNγ signaling pathways, activation of T cells, activation of dendritic cells, and response of T-helper type (Th1) cells, while the same was not shown in the other group donor [42]. Very similar research was conducted by Davar et al. in 2021, including 16 patients with anti-PD-1 refractory melanoma, who received FMT from seven donors previously exhibiting a sustained clinical response to anti-PD-1 therapy. Following the FMT six patients exhibited R, three patients had PR, and three patients achieved SD for more than 12 months. The composition of the microbiota in all recipients changed after FMT. Only half of the NR group had a microbiota composition similar to their donors, compared to all patients in the R group. In group R, the amount of *Firmicutes* (*Lachnospiraceae* and *Ruminococceaceae*) and *Actinobacteria* (*Bifidobacteriaceae* and *Corinobacteriaceae*) increased significantly, while the amount of *Bacteroidetes* decreased. In group R, higher amounts of activated and differentiated CD8+ T cells were found, and decreased amounts of circulating IL-8 systemically and intratumorally. Changes in circulating cytokines and chemokines were observed in R and NR, but more significantly in R [12].

On the other hand, Routy et al. conducted similar research with FMT on 20 patients with advanced melanoma but using the healthy subjects as donors, in contrast to Baruch and Davar. OR was demonstrated in 13 patients (group R), of which four patients achieved CR, and nine patients achieved PR. Within the NR group, two showed SD longer than 6 months, one SD shorter than 6 months, and four patients PD. Implantation was successful in all recipients, but after 1 month, the composition of the microbiota returned to that similar to that before FMT in the NR group, while in the R group, even after 6 months, it did not return to the initial composition but developed more similarity to the donor microbiota. The R group showed an increased amount of *Eubacterium ramuleus*, *Eubacterium siraeum*, and *Ruminococcus callidus*, and a decreased amount of *Enterocloster boltae* compared to the NR group. Of the intratumoral changes, the majority of R patients showed a greater infiltration of CD8+ T cells, but three R patients had one of the smallest infiltrations with these cells. The R group reported a significantly higher level of activation of CD8+ cells [43].

Only two FMT trials were conducted in non-melanoma patients. Kim et al. included four recipients with GC, five with ESCC, and four with HCC, all of which showed resistance to anti-PD-(L)1 inhibitors. FMT donors were chosen if they had SD or PR for at least 1 year with immunotherapy, and they were matched with the recipients based on the type of cancer. A total of 13 recipients received FMT. One patient exhibited PR, five achieved SD, and seven did not show a response. The recipients were treated with amoxicillin–clavulanate for 5 days before receiving their first FMT. *Prevotella merdae Immunoactis* was related to a positive response to immunotherapy, along with a CD8+ cell increase and CD4+ Treg decrease with increased IFN-γ secretion, while *Lactobacillus salivarius* and *Bacteroides plebeius* showed decreased IFN-γ secretion and immunosuppressive activity, inhibiting T-cell activation [44].

The most recent research on FMT and cancer immunotherapy was presented by Elkrief et al. in July 2024, who, along with melanoma patients, included 20 patients with advanced NSCLC and 20 with advanced melanoma refractory to immunotherapy. These patients received a one-time FMT after laxative bowel cleaning from one of 11 healthy donors, after which NSCLC patients were treated with dual immunotherapy. In the NSCLC group, no patients achieved CR, although 15 patients exhibited PR and 3 SD [45]. Data on FMT is summarized in Table 3.

## 2. Discussion

Although immunotherapy represents a major breakthrough in the treatment of cancer patients, its effectiveness needs to be increased, and a possible way to achieve this is by modulating the intestinal microbiota. However, early research in the field showed heterogeneous results in regards to providing the precise answer as to which microbiota is beneficial and which is detrimental for immunotherapy response. One of the main issues with evaluating the detrimental and beneficial microbiota is the heterogeneity of the trials with variable and often contradictory results. One of the potential explanations is the need to evaluate the microbiome as a dynamic prognostic factor and include a time variable in evaluations. McCulloch et al. showed that for melanoma patients, the early responses to treatment are primarily driven by intrinsic tumor and host factors, while the microbiota influence becomes most significant around 9 to 13 months into treatment, after which its effect may begin to decline. They indicated that shifts in the microbiome composition over time during immunotherapy treatment could contribute to treatment effectiveness, meaning less effectiveness of immunotherapy if certain beneficial bacteria become less dominant over time and non-immunogenic tumor factors begin to drive disease progression [18]. Similar results were shown by Lee et al., showing that the microbiota and immunotherapy connection is complicated and depends on demographics and cancer-specific characteristics [9], and by Golčić et al., who demonstrated a difference in microbiome and dietary patterns based on the time required for complete response during immunotherapy for advanced melanoma. Patients who exhibited a late response ingested more flavones and less protein and sweets, with a greater microbiota diversity [46].

Intestinal microbiota can affect the effectiveness of immunotherapy through the secretion of metabolites and by enhancing the innate and adaptive immune response to tumor cells. There are several ways in which it is possible to modulate the composition of the intestinal microbiota. One of the most common ways is through ABs. However, all the research on the relationship between AB and immunotherapy was retrospective. Furthermore, there are methodological differences as some authors focused on the timing of AB administration, while others tried to quantify AB doses, through DOT, DDD, AE, and differentiating the length of administration of AB longer or shorter than 7 days. While the majority of studies demonstrated a negative relationship between AB and the effectiveness of immunotherapy, others emphasize the timing, dose, and choice of AB, and potentially the type of cancer. Current data suggests that taking AB before the start of immunotherapy, having a higher amount of exposure to AB, and using broad-spectrum ABs showed the most negative impact on the effectiveness of immunotherapy. However, due to a lack of prospective studies, methodological difficulties, and other contributing factors, it remains difficult to determine exactly which AB and to what extent they are harmful. Nonetheless, rational use of AB is paramount, and a special concern must be attributed to cancer patients treated with immunotherapy.

There is much less research on the influence of probiotics and dietary habits on the effectiveness of immunotherapy, partially due to an unregulated market, differences in the types of probiotics available, and the previously mentioned methodological difficulties. Lack of sponsorship and funding for trials that do not involve a particular medicinal product, such as diet, further complicates the undertaking of any large clinical trial. Additionally, dietary questionnaires rely on patients’ memory, in contrast to studies related to AB, which obtain information about them from official medical records. While there is some data suggesting that some probiotics, such as *Clostridium butyricum* or LGG, could be beneficial in cancer patients treated with immunotherapy, Spencer et al. [39] demonstrated a negative association between probiotic use and survival and immune response. Hence, at this time, there are no suggestions for our patients for probiotic supplementation during immunotherapy outside clinical trials.

A diet with a high content of dietary fiber has been shown to correlate positively with the effectiveness of immunotherapy in various other trials. While more data is needed, the high-fiber diet is generally considered healthy if there are no medical contraindications and could be suggested to patients as a simple method to improve the response to immunotherapy even without substantial confirmatory data. Another dietary component that could be useful for immunotherapy response is salt, although the prospective data are scarce. However, several analyses in both mice and human patients did suggest a potentially positive correlation. However, using HSD is associated with serious health concerns and cannot be routinely suggested for patients outside clinical trials.

Additionally, although all FMT trials published so far have shown positive outcomes, there is a lack of standardization in methodology, the number of treatments, the number and the choice of donors, and adjuvant cancer treatment. However, the safety of FMT with immunotherapy was proven by the fact that out of a total of 98 patients, only 22 had grade three side effects, and there were no higher-grade side effects. A total of 62 out of 98 patients showed an objective clinical response, including 10 CR, 40 PR, and 12 SD. It is also important to emphasize that all five studies involved patients who showed primary or secondary resistance to the same immunotherapy with which they were treated as part of the study. These results indicate that FMT is a potential adjuvant option in addition to immunotherapy in patients with advanced cancers, but larger and standardized studies are needed to confirm the results.

## 3. Conclusions

Intestinal microbiota is an important part of the human body, the composition of which can affect the effectiveness of immunotherapy. The use of ABs, especially those that are of a broad spectrum and/or used in a larger amount, causes dysbiosis of the intestinal microbiota, due to which the effectiveness of immunotherapy can be reduced. The use of commercial probiotics without evidence of intestinal dysbiosis has not yet been sufficiently tested to confirm its safety for cancer patients undergoing treatment with immunotherapy. A diet consisting of a sufficient amount of fiber is recommended, while a high salt diet content positively correlates with the success of immunotherapy but cannot be considered as a standard practice outside clinical trials. Additionally, FMT is a safe and potentially effective adjuvant option for the treatment of cancer patients with immunotherapy, but it is necessary to standardize treatment protocols and establish more clinical evidence supporting the success of this type of treatment.

## Figures and Tables

**Table 1:** Review of studies on AB use and immunotherapy effectiveness.

Study	Cancer Type	Total Patients	Patients Treated with AB	Analyzed Time Period of AB Use (Days)	Median PFS (Months)		Median OS (Months)		PFS Difference *p*-Value	OS Difference *p*-Value
					No AB	AB	No AB	AB		
Derosa et al. [24]	RCC	105	16	−30	7.4	1.9	30.6	17.3	<0.01	=0.03
	NSCLC	191	48		3.8	1.9	24.6	7.9	=0.03	<0.01
Geum et al. [28]	NSCLC	70	70	−30 and during treatment	/	/	/	/	=0.9	=0.014
Elkrief et al. [26]	Melanoma	64	10	−30	7.3	2.4	18.3	10.7	=0.01	=0.17
Mohiuddin et al. [27]	Melanoma	454	114	−90	/	/	43.7	27.4	/	=0.01
Routy et al. [17]	NSCLC, RCC, urothelial	180	69	From −60 to +30	4.1	3.5	11.5	20.6	=0.017	<0.001
Iglesias-Santamaria et al. [29]	Melanom, a NSCLC, RCC, urothelial, other	60	42	From −28 to +28 or during treatment	5.8	4.4	13.3	13.8	=0.1	=0.1
Pinato et al. [25]	Melanoma, NSCLC, other	167	29	−30	/	/	26	2	/	<0.001
Tinsley et al. [30]	Melanoma, NSCLC, RCC	199	92	From −14 to +42	6.3	3.1	21.7	10.4	=0.003	=0.002
Glitza et al. [31]	Melanoma	6	8	From −7 to −3	15.0	5.2	/	21.1	>0.05	>0.05

Sign “−” = days before the start of immunotherapy, sign “+” = days after the first dose of immunotherapy, sign “/” = information not specified or not valid.

**Table 2:** Review of studies on probiotics and dietary habits and immunotherapy effectiveness.

Study	Cancer Type	Type of Modulation	Patients without Modulation	Patients with Modulation	Median PFS (Relative Compared to Other Group)		Median OS (Relative Compared to Other Group)		Remark
					No Modulation	Modulation	No Modulation	Modulation	
Tomita et al. [35]	NSCLC	*Clostrydium butiricum*	79	39	↓	↑	↓	/	Median OS not reached
Dizman et al. [36]	RCC	*Clostrydium butiricum*	10	19	↓	↑	/	/	Median PFS/OS not reached
Takada et al. [38]	NSCLC	Probiotics	32	262	↓	↑	=	=	Difference in OS not statistically relevant
Spencer et al. [39]	Melanoma	Probiotics	109	49	↑	↓	/	/	Median PFS/OS not reached
		Fiber intake	37	91	↓	↑	/	/	
		Probiotics + fiber intake	22	101	↑	↓	/	/	

OS and PFS are not expressed in absolute numbers because there is a big difference in the way the results of the studies are expressed, and in many of them, the median OS does not exist because most patients were alive when the last information was taken. Sign “/” = information not specified or not valid, sign “=” = no significant difference. Only human studies without Simpson, McCulloch, Lee, and Golčić are included in the table due to a different description of the results.

**Table 3:** Review of studies on FMT and immunotherapy effectiveness.

Study	Cancer Type	Donors	Recipient	FMT	Pre-FMT Treatment	Clinical Response				FMT Adverse Effects		Immunotherapy Adverse Effects	
						CR	PR	SD	PD	Gr. 1–2	Gr. 3–4	Gr. 1–2	Gr. 3
Baruch et al. [42]	Melanoma	2	10	1 colonoscopy + oral capsule	Vankomicine + Neomicine	1	2	0	7	1	0	/	0
Davar et al. [12]	Melanoma	7	15	1 colonoscopy	/	1	2	3	9	/	/	15	3
Routy et al. [43]	Melanoma	3	20	1 oral capsule	Laxative	4	9	1	5	8	0	12	5
Kim et al. [44]	GC, ESCC, HCC	6	13	1–4 colonoscopies	Amoxicilin clavulanate	0	1	5	7	/	/	6	1
Elkrief et al. [45]	NSCLC	11	20	1 oral capsule	Laxative	0	15	3	2	15			
	Melanoma		20			4	11	0	5	19			

Sign “/” = correct information is not provided or is not valid. Only human studies are included in the table.
